# Golden Syrian Hamsters as a Model for Revisiting the Role of Biological Sex Differences in SARS-CoV-2 Infection

**DOI:** 10.1128/mBio.01848-21

**Published:** 2021-11-23

**Authors:** Rafael Tomoya Michita, Indira U. Mysorekar

**Affiliations:** a Department of Medicine, Section of Infectious Diseases, Baylor College of Medicine, Houston, Texas, USA; b Department of Molecular Virology and Microbiology, Baylor College of Medicine, Houston, Texas, USA

**Keywords:** COVID-19, SARS-CoV-2, sex difference, Syrian hamster, lung infection, viral infections

## Abstract

There is growing evidence that coronavirus disease 2019 (COVID-19) affects males more severely than females, including compelling evidence indicating that biological sex is an important clinical factor influencing disease pathology and outcomes. In their recent article in *mBio*, S. Dhakal, C. A. Ruiz-Bedoya, R. Zhou, P. S. Creisher, et al. (mBio 12:e00974-21, 2021, https://doi.org/10.1128/mBio.00974-21) find further evidence to support this hypothesis as they interrogate biological sex differences in the pathogenesis and clinical features of COVID-19 in the golden Syrian hamster model. Their study probes SARS-CoV-2 infection in terms of loss of body mass, recovery, lung compromise, viral replication, inflammatory response, immune response, and, most importantly, the role of estrogen. They also demonstrate the value of a novel unbiased, quantitative chest computed tomography (CT) imaging approach. The golden Syrian hamster model holds a promising opportunity to further investigate how biological sex acts as a primary determinant in SARS-CoV-2 pathogenesis, as also demonstrated in this study.

## COMMENTARY

Since the first reports of SARS-CoV-2 infections from Wuhan, China, in December 2019, the spread of infections quickly reached a pandemic scale that has so far taken more than 4.6 million lives and continues to affect the human population globally. Although vaccination efforts have reduced fatal outcomes, the slow pace of vaccination at a global scale, especially in low- and middle-income countries, is concerning (https://covid19.who.int/). In addition to key features of SARS-CoV-2 biology, scientists have unraveled fundamental factors influencing host susceptibility to severe outcomes. Several reports have shown variable disease outcomes according to demographics and preexisting comorbidities (1, 2). Sex differences and associated risk behaviors are traditionally suggested as primary factors underlying the increased risk in men compared to women (3). This notion has often been evoked to explain sex differences in the biological study of diseases and outcomes, with remarkably limited or little consideration to the contribution of the biological sex *per se*. In the ongoing pandemic, the observed disease severity and slow recovery in males bring biological sex into the spotlight, calling attention to its underestimated impact in (but not limited to) SARS-CoV-2 infection (4).

Evaluating biological sex differences in COVID-19 is challenging, partly due to ethical issues and the inherent biological and cultural diversity of human populations (3, 5). Multiple studies have proposed animal models for studying diverse aspects of SARS-CoV-2 infection (6–9). Golden Syrian hamsters (Mesocricetus auratus) are an animal model possessing high sequence homology with the human ACE2 receptor. They are naturally susceptible to SARS-CoV-2 infection and present symptoms that resemble COVID-19 and vary according to biological sex and other demographic differences, such as age (10–12). Of note, it has been shown experimentally that the extension of lung disease and disease severity is male sex dependent in golden Syrian hamsters through a yet-unknown mechanism (13). In an attempt to investigate the question of biological sex as a factor with COVID-19, Dhakal et al. (14) have provided important insights by using the golden Syrian hamster. In their study, several clinical findings resembled those observed in previous COVID-19 studies in humans (viral replication, cytokine storm, antibody response, and respiratory compromise). This supports use of the golden Syrian hamster as a *de facto* standard model for studying COVID-19.

The study showed that viral replication in the respiratory tract was similar between sexes, establishing an equal susceptibility to SARS-CoV-2 infection in controlled settings. Morbidity was assessed systemically in terms of body mass and locally in terms of pulmonary damage. Loss of body mass was significantly greater in males (−17.3 ± 1.9%) than in females (−12.3 ± 1.8%) after 8 to 10 days postinfection (dpi), with recovery to baseline body mass taking up to 3 weeks for males compared to 2 weeks for females. Uniquely, the authors provided a novel unbiased chest computed tomography (CT) scoring method to evaluate respiratory compromise with potential application to other animal models. In this study, lower CT scores of pulmonary consolidation were observed in females compared to males. This method for quantitative CT imaging analysis is a promising tool for evaluating lung disease that can reduce bias in visual assessment and improve the reproducibility of results in future studies.

The authors go a step further and evaluate how estradiol (E2) treatment in males affects SARS-CoV-2 disease severity. Sexual hormones such as E2 have been implicated in enhancing the immunologic and humoral response phenotype in females during viral infections (reviewed in reference 15). Strikingly, E2 administration in gonadally intact male hamsters before infection did not improve morbidity outcomes (body mass loss, CT score, and histology findings) compared to placebo-treated males. This suggests that E2 administration for a short period is insufficient for replicating the expected long-lasting effects of female hormones in males. Besides the dual effect of high and low E2 concentrations, the type of receptor and distribution among cell types are additional factors to be considered (16). Considering that E2 levels in treated males reached up to ∼150 pg/ml, an important topic of future investigation will be how the estrous cycle (30 to 700 pg/ml) in golden Syrian hamsters affects the SARS-CoV-2 response. This also raises questions for further investigation about the role of other sex-related hormones, particularly testosterone, as the authors noted. Also, SARS-CoV-2 has been detected in the testes of infected golden Syrian hamsters and may be important to understanding fundamental aspects of short- and long-term impact of COVID-19 in male reproductive health (17).

**FIG 1:**
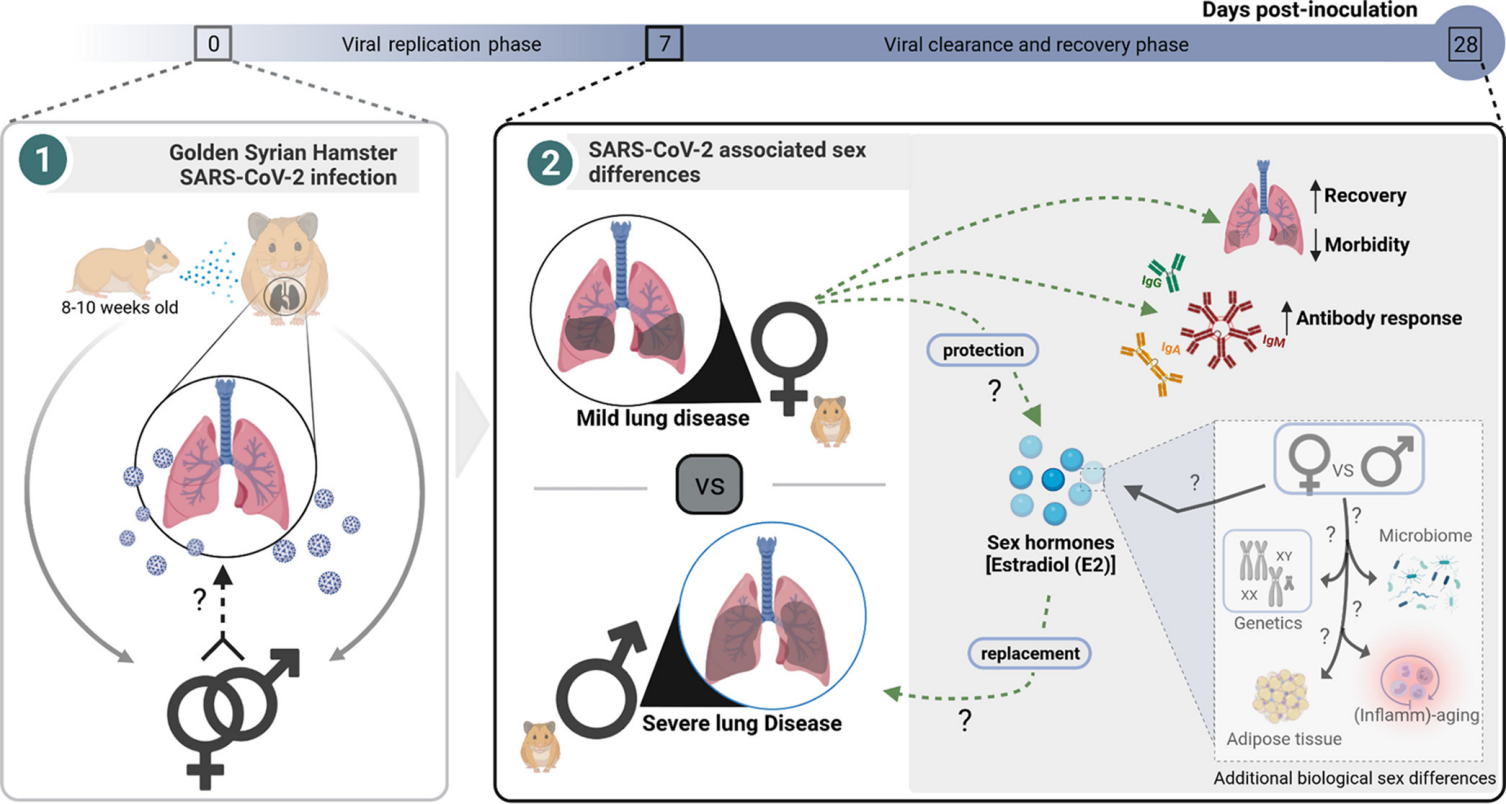
Biological sex influences SARS-CoV-2 infection outcomes in golden Syrian hamsters. Dhakal and colleagues describe a model for SARS-CoV-2 infection in young adult hamsters and demonstrate that male and female animals are equally susceptible to infection in initial stages of infection. Interestingly, however, female hamsters show lower morbidity, developing less extensive pneumonia, and greater antibody response to SARS-CoV-2 than male hamsters. Differences in sex hormone, estradiol (E2), may drive the differential susceptibility, although exogenous addition of E2 does not reverse the male susceptibility (14).

Similarities with the human inflammatory response to SARS-CoV-2 further support the use golden Syrian hamsters to investigate aspects of the immune response (e.g., cytokine storm and type I interferon [IFN] response) in COVID-19. Although the study showed most cytokine lung homogenate levels were similarly higher in infected animals for both sexes, only males showed higher lung *Tnfa* expression and protein levels in the first days postinoculation (lung at 2 dpi) correlating with viral load. Considering biological sex differences, it has been shown that *Tnfa* increases the conversion of local and systemic androgens to estrogens through aromatase in inflammatory conditions (reviewed in reference 18), which may increase the humoral response and the proliferation of antigen-presenting cells. A relevant topic for future investigation will be whether androgen conversion to estrogen (a known risk for autoimmunity) also occurs in golden Syrian hamsters and if resulting stimuli could give clues to the rise of autoantibodies (type I interferons) observed in COVID-19 severity and recovery (19). Hence, the measurement of sexual hormones will provide insights of how they affect the course of disease. Most notably, Dhakal et al. provide evidence that cytokine levels have a limited correlation with sex differences and that differences in disease outcomes are influenced primarily by biological sex through another mechanism or combination of mechanisms (Fig. 1).

These authors also showed compelling evidence that females mount a higher and broader humoral response than males during SARS-CoV-2 infection. The study showed this with IgM, IgA, and IgG antibody responses against the receptor-binding domain of the spike protein (S-RBD). Females also showed a greater cross-reactive antibody response to SARS-CoV-2 S-RBD variants (anti-S-RDB IgG, wild type, N501Y, Y453F, N439K, and E484K). Similar results were also observed at the tissue level. It will be of interest to further evaluate whether androgen treatment influences humoral response and priming in females and whether the infectiousness of emerging SARS-CoV-2 variants of concern is sex biased.

Broadly speaking, this is an exciting study that further demonstrates how key clinical features resembling COVID-19 are observed in golden Syrian hamsters upon SARS-CoV-2 infection and how those features can vary by sex. The results of the study are intriguing and contribute to many questions yet unanswered of how biological sex influences SARS-CoV-2 infection outcomes in golden Syrian hamsters with clear implications for human COVID-19. The key to understanding this phenomenon starts with recognizing that biological sex transcends its usual classification as a demographic variable, and sex-associated features have distinct contributions in healthy and pathological conditions. These features include the microbiome, hormonal changes, metabolism (adipose tissue distribution), behavior, and genetics (X-chromosome genes such as *TLR7* and *ACE2* and related haploinsufficiency in males) (20, 21). The implications of this study by Dhakal et al. extend to other fields of biology, as well as medicine and epidemiology, and may foster comparative analysis, primarily on infections, but also on a wide range of diseases to help understand and increase the reproducibility of results among studies.

Potential mechanism(s) underlying the biological sex differences that need to be investigated include genetics (X-inactivation, genetic variants, and epigenetics), microbiome (metabolomics), sex hormones (androgen, estrogen, and estrous cycle), metabolism (biological sex differences in adipose tissue distribution, visceral and subcutaneous), and aging.
